# Quinacrine promotes autophagic cell death and chemosensitivity in ovarian cancer and attenuates tumor growth

**DOI:** 10.18632/oncotarget.5632

**Published:** 2015-10-16

**Authors:** Ashwani Khurana, Debarshi Roy, Eleftheria Kalogera, Susmita Mondal, Xuyang Wen, Xiaoping He, Sean Dowdy, Viji Shridhar

**Affiliations:** ^1^ Department of Experimental Pathology, Mayo Clinic College of Medicine, Rochester, MN, USA; ^2^ Department of Obstetrics and Gynecology, Mayo Clinic College of Medicine, Rochester, MN, USA

**Keywords:** quinacrine, ovarian cancer, autophagy, apoptosis and tumorigenesis

## Abstract

A promising new strategy for cancer therapy is to target the autophagic pathway. In the current study, we demonstrate that the antimalarial drug Quinacrine (QC) reduces cell viability and promotes chemotherapy-induced cell death in an autophagy-dependent manner more extensively in chemoresistant cells compared to their isogenic chemosensitive control cells as quantified by the Chou-Talalay methodology. Our preliminary data, *in vitro* and *in vivo*, indicate that QC induces autophagy by downregulating p62/SQSTM1 to sensitize chemoresistant cells to autophagic- and caspase-mediated cell death in a p53-independent manner. QC promotes autophagosome accumulation and enhances autophagic flux by clearance of p62 in chemoresistant ovarain cancer (OvCa) cell lines to a greater extent compared to their chemosensitive controls. Notably, p62 levels were elevated in chemoresistant OvCa cell lines and knockdown of p62 in these cells resulted in a greater response to QC treatment. Bafilomycin A, an autophagy inhibitor, restored p62 levels and reversed QC-mediated cell death and thus chemosensitization. Importantly, our *in vivo* data shows that QC alone and in combination with carboplatin suppresses tumor growth and ascites in the highly chemoresistant HeyA8MDR OvCa model compared to carboplatin treatment alone. Collectively, our preclinical data suggest that QC in combination with carboplatin can be an effective treatment for patients with chemoresistant OvCa.

## INTRODUCTION

Ovarian cancer remains a leading cause of death among women with gynecological cancers despite significant advances in the systemic as well as surgical cancer treatment modalities [1]. Ovarian cancer patients with advanced or recurrent disease frequently develop chemoresistance against paclitaxel- and platinum-based therapies which further contributes to disease progression, recurrence and, ultimately, high mortality [2]. In order to overcome the shortcomings of standard chemotherapeutic modalities, several alternative strategies including targeted therapies, multi-drug combination treatments as well as drug repurposing are being investigated. Although the majority of these therapeutic compounds induce apoptosis through type I programmed cell death (PCD), compounds inducing type II PCD have also been shown to be effective as anti-cancer agents [3]. Predominant feature of type II or autophagic cell death is the appearance of double-membrane vesicles engulfing cytoplasmic organelles which are eventually degraded by lysosomal hydrolytic enzymes. Extensive autophagic activity leads eventually to cell death which, however, differs from the homeostatic autophagy associated with normal cellular organelle turnover [4]. Under basal cellular conditions, autophagy maintains the cellular turnover of proteins and organelles via lysosomal degradation whereas, under nutrient-deprived stress conditions such as oxidative and/or endoplasmic reticulum stress [5], it promotes cellular adaptation by supplying macromolecules for survival [6]. Various strategies have been investigated to explore the potential of autophagy as a putative anticancer modality including development of chemical inhibitors of autophagy as well as genetic silencing of key autophagy proteins [7]. Several studies have shown that inhibiting autophagy using anti-malarial compounds such as chloroquine (CQ) and hydroxychloroquine while combining these compounds with frontline therapeutic agents such as cisplatin and taxol results in significant inhibition of tumor growth [8, 9]. Furthermore, other studies have indicated that drug-induced autophagy promotes synergy with the frontline therapy [10, 11]. Similarly it has been shown that genetic silencing of key autophagic proteins such as beclin 1 (ATG6) favors survival and decreases resistance to chemotherapy [12–14]. High beclin 1 and LC3 levels in ovarian tumors have been associated with improved overall survival [15, 16].

The two most common markers and key players associated with autophagy are LC3B and p62 [17]. Events leading to the conversion of LC3BI to LC3BII and clearance of p62 are considered hallmarks of the autophagic flux [18]. The p62 protein, also called sequestosome 1 (SQSTM1), is an ubiquitin-binding scaffold protein that co-localizes with ubiquitinated protein aggregates and is required both for the formation as well as the degradation of polyubiquitin-containing bodies by autophagy. p62 binds to LC3B through the LIR (LC3 Interacting Region) domain and is then degraded during the autophagic process [19]. Other studies have shown that elimination of p62 by autophagy suppresses tumorigenesis [20] *in vivo* and cell viability of several human carcinoma cell lines *in vitro* [21]. Since p62 accumulates when autophagy is inhibited, and alternatively, p62 levels decrease when autophagy is induced, p62 surfaces as a promising marker to study autophagic flux. Selective degradation of p62 is clinically relevant since high levels of p62 found in various types of tumor have been associated with poor prognosis and survival [22]. Studies show that the cisplatin-resistant SKOV3/DDP OvCa cells express higher levels of p62 and that siRNA downregulation of p62 in these cells resensitized them to cisplatin-mediated cytotoxicity [23].

Previous investigations have provided evidence to suggest a promising role of the antimalarial drug quinacrine (QC) in cancer treatment. The acridine “backbone” of QC allows the drug to intercalate into stacked DNA base pairs [24]. QC is known to impair DNA repair activity in a mechanism similar to other topoisomerase inhibitors, [25]. In addition, QC inhibits the FACT (Facilitates Chromatin Transcription) complex that is required for NF-kB transcriptional activity and modulates the arachidonic acid (AA) pathway [26]. Interestingly, QC has been shown to bind and inhibit proteins involved in multidrug resistance [27–32]. More importantly, it targets several signaling pathways simultaneously by affecting autophagy, apoptosis, p53, NFkB, AKT and methylation-related pathways [27, 28, 32–35]. While QC has been shown to modulate autophagy in a p53-dependent manner in colon cancer cell lines, [36] in our study QC induced autophagic cell death in a p53- independent manner in OvCa cells. Although QC has been shown to effectively block proliferation of several cancer cell lines both *in vitro* and *in vivo*, to our knowledge, there are no *in vitro* or *in vivo* studies on the use of QC alone or in combination with standard therapy against OvCa.

In this study, we have shown that QC promotes autophagic flux across a variety of OvCa cell lines and induces cell death both in a caspase-dependent as well as independent manner utilizing autophagic-mediated cell death to enhance carboplatin sensitivity. This effect was more pronounced in cisplatin-resistant OvCA cells compared to their sensitive controls both *in vitro* and *in vivo* experimental setting. These preclinical data have direct clinical implications for OvCa patients with chemoresistant disease for which only limited therapeutic options exist.

In this study, we focused our investigation on the anticancer potential of the antimalarial drug QC against OvCA. Based on prior findings, we hypothesized that QC would exert its anticancer effect against OvCA by inducing an autophagic-mediated cell death and that by doing so it would result in restoring cisplatin-sensitivity.

## RESULTS

### Quinacrine inhibits cell growth and induces cell death in ovarian cancer cells

**Figure 1:**
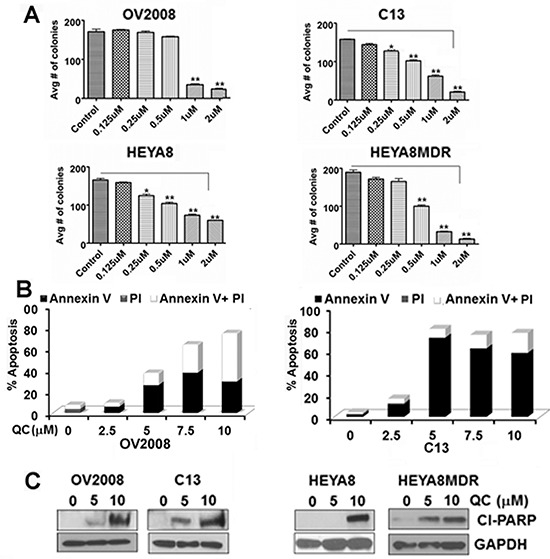
**A.** OV2008, C13, HeyA8 and HeyA8MDR cells plated in six well plates in triplicates were treated with indicated concentrations of QC for 24 hours The resulting colonies were counted after fixing and stained with crystal violet in methanol and photographed. Data are representative of three independent experiments. P values were calculated using student t test. *p value = < 0.001, **p value = < 0.0001 **B.** AnnexinV/Propidium Iodide (PI) staining of OV2008, C13, HeyA8 and HeyA8MDR cells treated with Quinacrine for indicated concentration for 24 hours. **C.** Immunoblot analysis of cell lysates obtained from OV2008, C13, HeyA8 and HeyA8MDR cells treated with Quinacrine (0, 5.0 μM, 10.0 μM) for 24 hours with anti-cleaved PARP and anti-GAPDH antibodies. Data are representative of three independent experiments.

Isogenic pairs of OvCA cell lines [OV2008 (chemosensitive) and C13 (chemoresistant) cells derived from OV2008 [37]; HEYA8 (chemosensitive) and HEYA8MDR (chemoresistant) [38, 39] cells] were evaluated for the effect of QC on cell growth by colony formation and MTT assays. Colony formation assays (Figure 1A) were performed after treating the cells with 0, 0.125, 0.250, 0.500, 1.0, and 2.0 μM of QC for 24 hours. MTT assays (supplemental data) were performed after treating the cells with 0, 5.0, and 10.0 μM of QC for 24, 48 and 72 hours-time intervals. Increasing concentrations of QC effectively inhibited colony forming units with maximal inhibition at a QC concentration of 1.0 and 2.0 μM. Similarly, cell growth was also inhibited as early as 24 hours of QC treatment with IC_50_ determined from the MTT assays in all the cell lines tested were between 2.5 μM and 4 μM (Figure supplemental S1). To determine if QC treatment induced apoptotic cell death, we treated cells with 2.5, 5.0 and 7.5 μM QC for 24 hours and the apoptotic cell population was determined with the annexin/PI staining method using flow cytometric analysis. The apoptotic cell population upon QC treatment reflecting early and late apoptosis as shown in Figure 1B indicates that QC only treatment induces apoptosis. Similarly, western blot analyses of cell lysates of OV2008/C13 and Hey A8/HeyA8MDR cells treated with 5.0 and 10 μM QC showed the presence of cleaved PARP corroborating the previous finding that QC promotes apoptosis in a caspase-dependent manner (Figure 1C).

### Quinacrine induces autophagic clearance of p62/SQSTM, upregulates the autophagic marker LC3B and induces apoptosis

**Figure 2:**
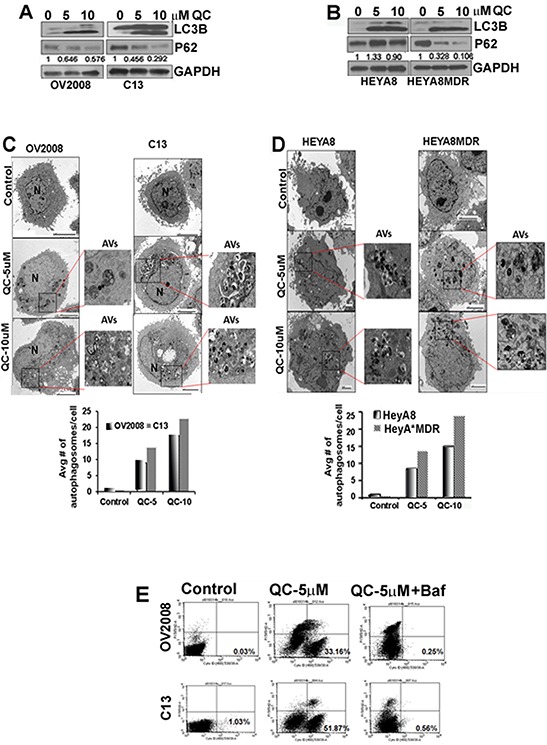
**A.** Immunoblot analysis of cell lysates obtained from OV2008, C13, **B.** HeyA8 and HeyA8MDR cells treated with Quinacrine (0, 5.0 μM, 10.0 μM) for 24 hours with anti-LC3B, p62 and anti-GAPDH antibodies. Values below the western blot panel indicate densitometry analysis of the blot. **C** and **D.** TEM analysis of QC treated OV2008, C13, HeyA8 and HeyA8MDR cells were performed to detect the presence of autophagic vesicles. Magnified inset shows double membrane autophagic vesicles in QC treated OV2008, C13 (C), HeyA8 and HeyA8MDR (D) cells. Quantitation of autophagy was estimated by counting the number of autophagic vesicles and results were plotted in bar graph. **E.** Autophagy was detected using cyto-ID fluorescence dye in OV2008 and C13 cells treated with QC in the presence or absence of bafilomycin A1 for 24 hours using flow cytometer. Percent of cyto-ID detection is indicated. Data are representative of three independent experiments.

Since other antimalarial drugs have previously been shown to modulate autophagy [36], we tested whether QC is able to induce autophagy in addition to promoting apoptosis. Towards this end, OV2008/C13 as well as HeyA8/HeyA8MDR cells were treated with 5.0 and 10.0 μM QC for 24 hours. Western blot analysis of QC-treated cell lysates was performed using two different autophagic marker proteins, LC3B and p62. Figures 2A and 2B show induction of the lipidated form of LC3B-II in all four cell lines upon QC treatment. However, QC treatment reduced p62 levels significantly more in the chemoresistant (C13 and HEYA8MDR) cells compared to their sensitive counterparts. This induction of LC3B-II (lower band) and degradation of p62 are considered hallmarks of autophagic activation [40, 41]. QC treatment resulted in similar effects on p62 and LC3B independent of p53 status in high grade serous cell lines such as OVCAR3 (p53 mutant) and CAOV3 (p53 null) (Figure Supplemental S2). To further evaluate QC-induced autophagy, we utilized transmission electron microscopy (TEM). TEM analysis of QC-treated OV2008, C13, HeyA8 and HeyA8MDR cell revealed early autophagic bodies (autophagosomes) harboring intact organelles [18] (Figures 2C and 2D). Quantitation of autophagosomes is shown next to the respective figures. These data suggest that QC treatment induces autophagy.

While TEM analysis revealed qualitative and morphological features of autophagy upon QC treatment, it was unclear whether this represented increased generation of autophagosome or inhibition of autophagosomal maturation [42]. Therefore, to further clarify whether QC induces autophagic flux, OV2008 and C13 cells were treated with QC in the presence or absence of a potent late-autophagy inhibitor, bafilomycin A1, and then stained with the autophagolysosome-specific dye cyto-ID. Cyto-ID retention in the cytoplasm was detected by flow-cytometric analyses (Figure 2E). This data indicates that QC's ability to promote autophagy can be completely inhibited by co-treatment with bafilomycin A1.

### QC induces autophagic flux and QC-induced autophagy precedes apoptosis

**Figure 3:**
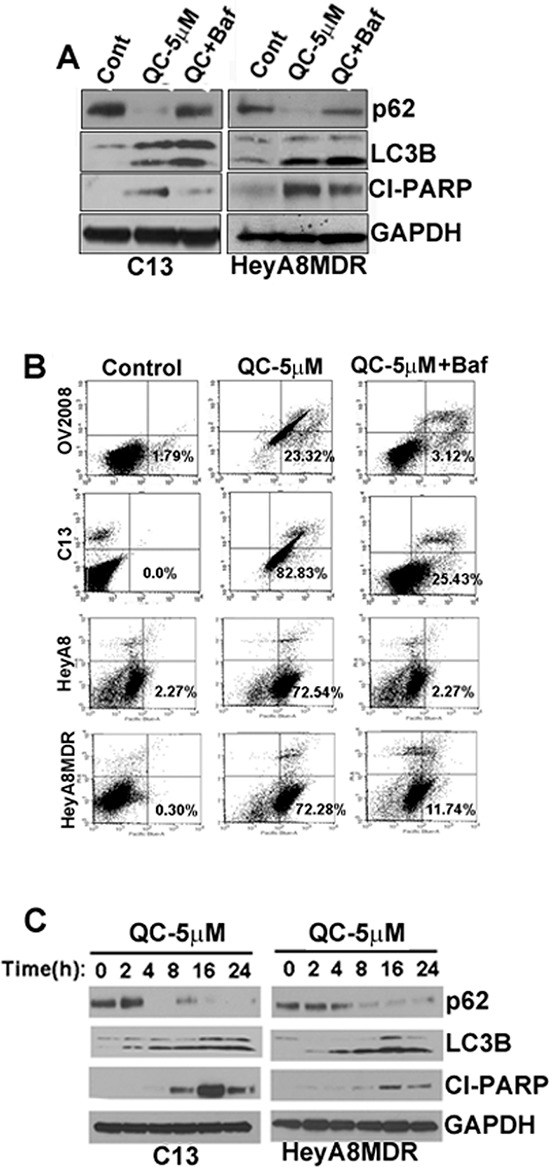
**A.** Autophagic flux was determined in C13 and HeyA8MDR cells Cells were either treated with QC and/or co-treated with bafilomycin A1 for 12 hours. Cell lysates were subjected to Immunoblot analysis using anti-p62, anti-LC3B, anti-cleaved PARP and anti-GAPDH antibodies. **B.** AnnexinV/Propidium Iodide (PI) staining of OV2008, C13, HeyA8 and HeyA8MDR cells treated with QC and/or co-treated with Bafilomycin A (100 nM) for 24 hours. Data are representative of three independent experiments. **C.** Immunoblot analyses were performed to detect the protein expression of p62, LC3B, cleaved PARP and GAPDH in C13 and HeyA8MDR cells after treating the cells for different time points (2, 4, 8,c16 and 24 hrs) with QC.

To further evaluate autophagic flux, we determined whether QC-mediated upregulation of LC3BII and downregulation of p62 was affected by co-treatment with bafilomycin A. Western blot analysis revealed that QC effectively downregulated p62 expression in C13 and HeyA8MDR and co-treatment with bafilomycin A protected autophagic p62 degradation (Figure 3A). No change in p62 mRNA levels was detected upon QC treatment (data not shown). We next tested whether QC-mediated apoptosis is dependent on autophagy. OV2008, C13, HEYA8 and HEYA8MDR cells were treated with QC with or without bafilomycin A1 followed by annexin/PI staining and flow cytometric detection of apoptotic cells. QC treatment in OV2008, C13, HeyA8 and HeyA8MDR cells resulted in varying but significant increase in annexin V positive cells. Co-treatment with bafilomycin A completely abolished QC-mediated annexin V positivity in these cells as shown in Figure 3B. Consistent with the flow cytometric data, immunoflourescence analysis also confirmed that the downregulated p62 by QC were rescued by bafilomycin treatment in C13 cells (Figure supplemental S3). This data indicates that QC-promoted cell death is completely reversed by autophagic inhibitors in all the four cell lines tested. Taken together, this data suggests that QC induces apoptosis in an autophagic-dependent manner.

Our data indicates that QC-induces apoptosis and autophagic inhibition by bafilomycin A. Therefore, we next sought to determine whether autophagy preceded the QC-induced apoptotic response. To this end, we determined the temporal regulation of autophagic proteins LC3B, p62 and apoptotic proteins such as cleaved PARP upon QC treatment at set time points in C13 and HeyA8MDR OvCa cells by western blot analysis. Data in Figure 3C indicate that QC treatment induced p62 downregulation and LC3B upregulation as early as 4 hours post treatment and these levels were sustained up to 24 hours following treatment. Detection of cleaved PARP was minimal at 4 hours of treatment; however its expression increased significantly at 16 hours post QC treatment. This data suggests that QC treatment triggered an autophagic response at the earlier time points while this autophagic response coincided with an apoptosis rate at later time points it.

### QC synergizes with carboplatin in ovarian cancer cell lines

**Figure 4:**
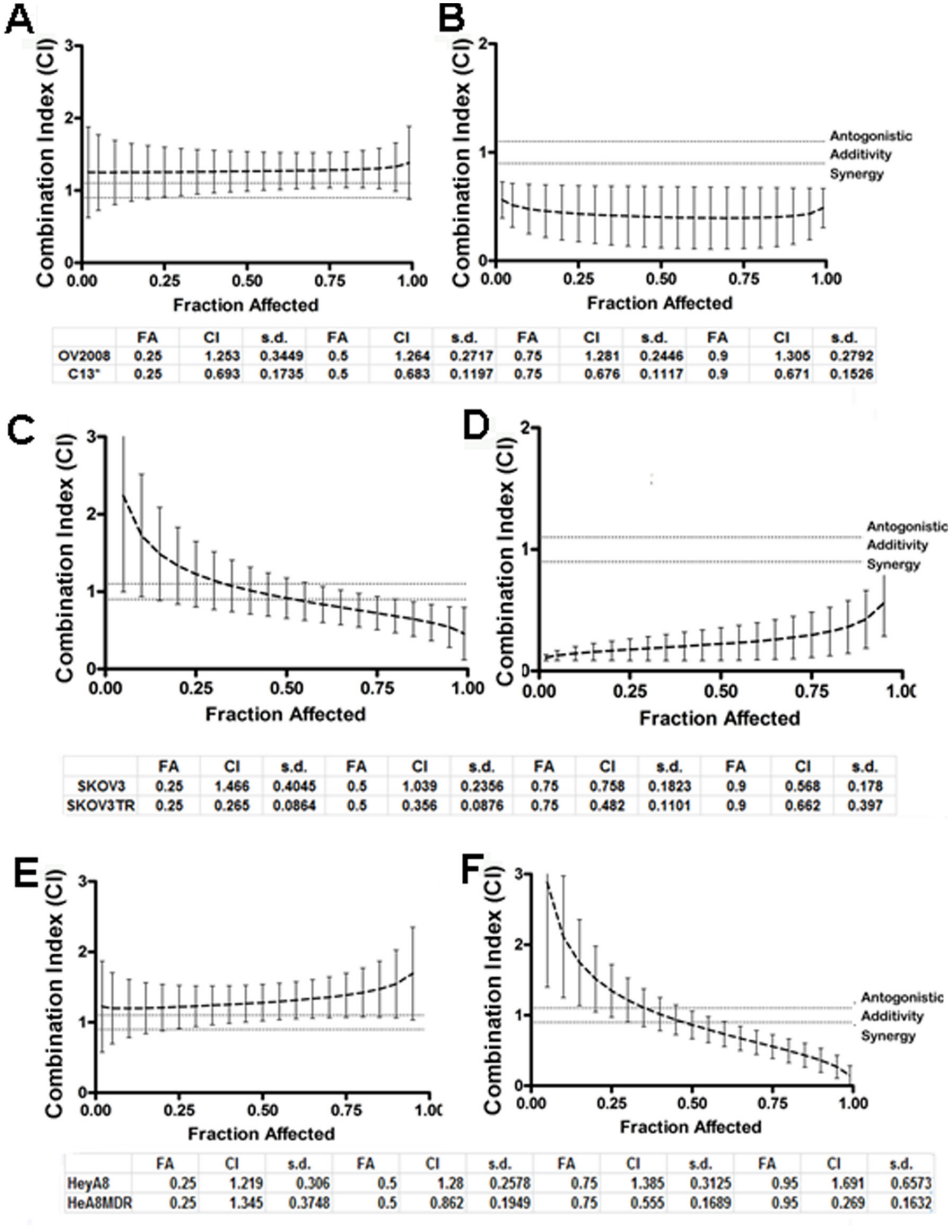
Quinacrine sensitizes ovarian cell lines to cisplatin-induced cytotoxicity Combination of quinacrine with cisplatin in equipotent combinations (IC_50_ over IC_50_ ratio) was assessed for synergy using the Chou-Talalay method. The cells were exposed to each drug alone and in combination per protocol for 48 hours. The combination indices (CI), fraction affected (Fa) in OV2008 and C13 **A** and **B.** in SKOV3 and SKOVTR **C** and **D.** and in Hey A8 and HeyA8MDR **E.** and **F.** were generated by the Calcusyn software and plotted with the use of GraphPad. Combination index (CI) values at 25, 50, 75 and 90% fraction affected (FA) are presented in the tables below the graphs. CI between 0.3–0.7 indicates strong synergism, 0.7–0.85 moderate synergism, 0.85–0.9 slight synergism, 0.9–1.10 nearly additive effect and greater than 1.10 antagonism.

After observing increased degree of apoptosis upon QC treatment in chemoresistant OvCa cells, we then sought to investigate whether QC can synergize with carboplatin treatment. In order to determine whether QC synergizes with cisplatin and carboplatin, constant ratio synergy studies were performed in isogenic cisplatin-sensitive OV2008 and cisplatin-resistant C13 cells by treating them with 1 × IC_50_ of cisplatin in combination with 1 × IC_50_ of QC. We observed that QC had a more potent synergistic anti-proliferative effect *in vitro* when combined with either cisplatin in C13 compared to OV2008 cells (Figures 4A and 4B). The combination indices (CI) for the corresponding fractions affected (FA) are shown in the tables below the figures. CI values between 0.1–0.3 indicate extremely strong synergism, 0.3–0.7 strong synergism, 0.7–0.85 moderate synergism, 0.85–0.9 slight synergism and 0.9–1.0 a nearly additive effect. Of note, synergy was demonstrated across nearly the entire range of the drug concentrations. Similar studies with isogenic taxol-sensitive SKOV3 and taxol-resistant SKOV3TR cells [39] indicate that QC has a more synergistic antiproliferative effect *in vitro* when combined with either cisplatin or carboplatin in SKOV3TR (Figures 4C and 4D) compared to SKOV3 cells. Consistent with this finding, chemoresistant HeyA8MDR cells showed stronger synergy when carboplatin was combined with QC compared to the parent chemosensitive HeyA8 cells (Figures 4E and 4F). Similar results were obtained with the combination of carboplatin and QC. Comparison of CI values for cisplatin and carboplatin are shown Figure Supplemental figure S4 in a tabular form for OV2002, C13, SKOV3 and SKOV3TR. The IC_50_ values for QC, cisplatin and carboplatin in all six cell lines used in synergy studies are shown in Fig S4. It is important to note that although the cell lines used in these studies are no longer considered representative of high grade serous cancers, they were initially chosen due to the availability of isogenic chemosensitive and chemoresistant pairs of OvCA cells.

### QC-induced autophagy is required for sensitization to cisplatin-mediated cell death in OvCa cells

**Figure 5:**
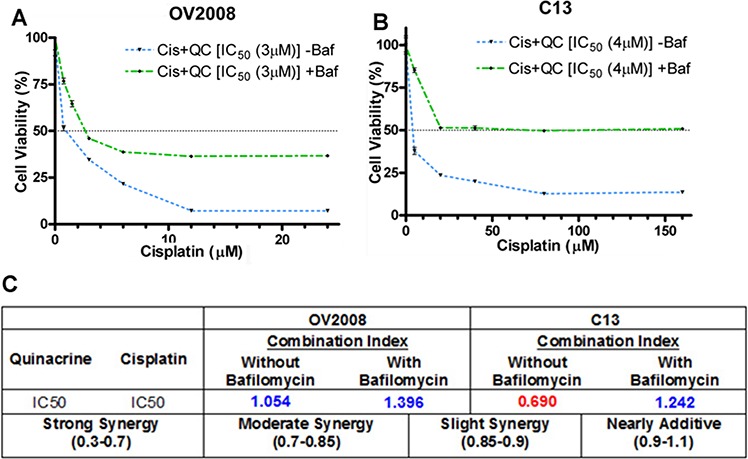
**A & B.** Quinacrine-induced autophagy is required for sensitizing cisplatin-mediated cell death of ovarian cancer cells Cell viability assays were performed with a combination of increasing concentrations of cisplatin with QC (1 × IC_50_) with and without bafilomycin pretreatment in OV2008 and C13 cells. Cells were pretreated with 50 nM bafilomycin for 2 hours followed by drug treatment. Cell viability was assessed by MTT assays 48 hours later. Pretreatment with bafilomycin (green dotted line) inhibited the combined QC plus cisplatin-induced cytotoxicity (blue dotted line) more effectively in C13 cells compared to OV2008. **C.** CI values of combination treatment without bafilomycin indicated strong synergy in C13 (*CI* = 0.690) and nearly additive effect in OV2008 (*CI* = 1.054), whereas, after bafilomycin pretreatment, CI values indicated antagonism in both C13 (*CI* = 1.242) and OV2008 (*CI* = 1.396).

In order to test whether QC-induced autophagy is essential in order to sensitize OvCa cells to cisplatin-induced cytotoxicity, we assessed the effects of the combination of increasing concentrations of cisplatin with QC (1 × IC50) with and without bafilomycin A pretreatment in OV2008 and C13 cells. We hypothesized that by inhibiting autophagy, bafilomycin A will inhibit the ability of QC to synergize with cisplatin in inducing autophagic cell death. The cells were pretreated with 50 nM of bafilomycin for 2 hours followed by treatment with cisplatin and QC. Cell viability was assessed by MTT assays 48 hours later. As shown in figures 5A and 5B, pretreatment with bafilomycin A (green dotted line) inhibited the combined QC- and cisplatin-induced cytotoxicity (blue dotted line). This is also demonstrated by the change in the CI values from values indicating strong synergy in C13 (*CI* = 0.690) and nearly additive effect in OV2008 (*CI* = 1.054) without bafilomycin to indicating antagonism in both C13 (*CI* = 1.242) and OV2008 (*CI* = 1.396) after bafilomycin pretreatment (Figure 5C). Collectively, these data suggest that QC-induced autophagy is necessary for resensitization to cisplatin-induced cell death in OvCa cells.

### p62 knockdown enhances sensitivity to carboplatin treatment in HeyA8MDR and C13 ovarian cancer cells

**Figure 6:**
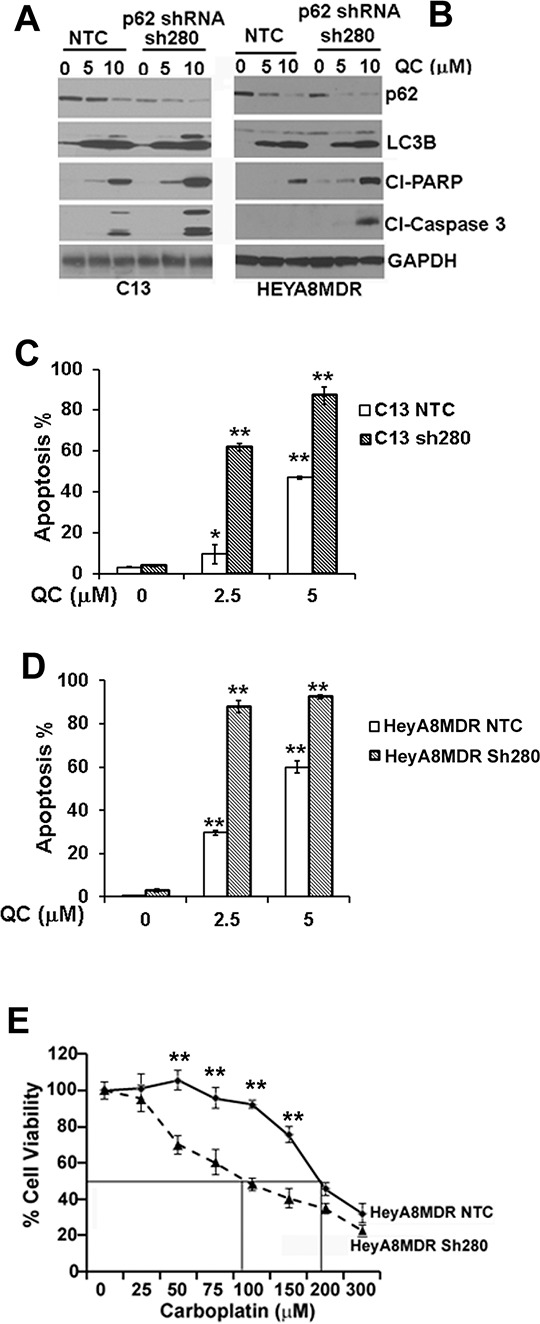
Immunoblot analysis of Non-targeted control shRNA (NTC) or p62 shRNA stable clones sh280 in A. C13 and B HeyA8MDR were treated with QC (0, 5.0 μM, 10.0 μM) for 24 hours with anti-cleaved PARP, cleaved caspase 3, p62, LC3BII and anti-GAPDH antibodies. **C & D.** AnnexinV/Propidium Iodide (PI) staining of OV2008, C13, HeyA8 and HeyA8MDR cells treated with Quinacrine for indicated concentration for 24 hours. Data are representative of two independent experiments. (*P = < 0.001, **P = < 0.0001). **E.** HEYA8MDR NTC and sh280 cells were treated with different concentrations of carboplatin and cell viability was measured by MTT assay. sh280 cells showed more sensitivity towards carboplatin treatment compared to the NTC group (**P < 0.0001, Figure 6E) indicating the critical role of p62 as a determinant of chemoresistance in HeyA8MDR cells.

Elevated levels of p62 have been previously shown to be critical in imparting chemoresistance in OvCA cells [43]. Our data indicate that QC treatment downregulated p62 levels preferentially in the chemoresistant C13 and HeyA8MDR cells (Figure 2A). Previous studies have shown that p62 downregulation sensitizes cells to cisplatin-mediated cytotoxicity [23]. To determine whether p62 plays role in QC- and carboplatin-mediated apoptosis, we generated two different p62 knockdown shRNA clones in HeyA8MDR and C13 cells as described in Materials and methods section with non-targeted control transduced cells (NTC) as controls. Efficient knockdown of p62 was confirmed in C13 and HeyA8MDR cells by western blot analysis using anti-p62 antibody (Figures 6A and 6B). To further evaluate the effect of QC in inducing apoptosis in NTC and p62-depleted cells, we treated C13 NTC and p62 shRNA clones with 0, 5.0, 10.0 μM of QC for 24 hours. Evaluation of apoptotic marker proteins by western blot analysis revealed that p62 knockdown cells exhibited higher degree of cleaved PARP and caspase 3 whereas no significant change was observed in LC3B II induction upon QC treatment in C13 NTC as well as p62shRNA cells (Figure 6A). Consistent with this data, QC treatment in HeyA8MDR p62shRNA cells showed increased degree of caspase 3 and PARP cleavage while no change was detected in LC3B II induction (Figure 6B). This data suggests that p62 downregulation in C13 and HeyA8MDR sensitizes the cells to QC treatment. Similarly, annexin/PI staining of these cells after treatment with QC revealed that C13 and HeyA8MDR p62 knockdown cells were more sensitive to QC-induced cell death when compared to NTC cells (Figures. 6C and 6D). More importantly, genetic downregulation of p62 in HeyA8MDR cells enhanced carboplatin sensitivity (please note the reduction in carboplatin IC50 from 176 μM in NTC cells to 110 μM in p62 knockdown cells) (Figure 6E). Collectively, these findings indicate that p62 downregulation sensitizes cells to QC-mediated cell death.

### Quinacrine synergizes with carboplatin in reducing HeyA8MDR derived mouse tumor xenografts

**Figure 7:**
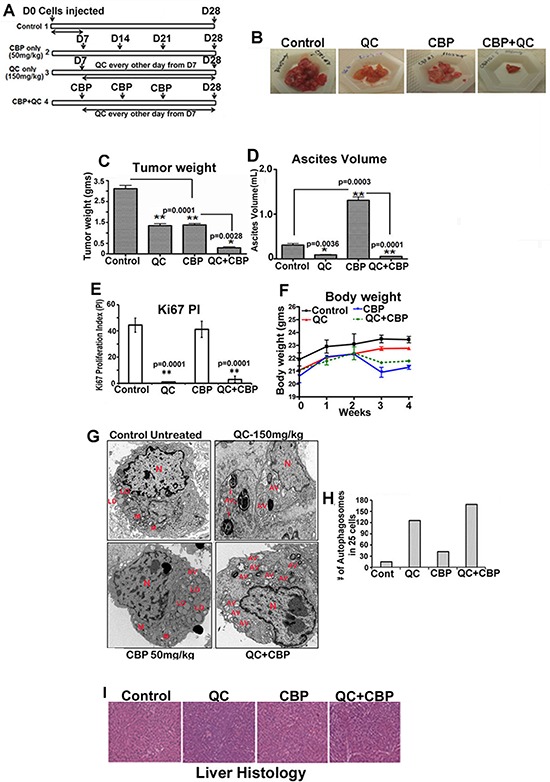
Quinacrine inhibits ovarian tumor growth *in vivo* A Schematic representation of treatment plan **B.** Representative tumors obtained from vivisected mouse showing HeyA8MDR tumors from control untreated, CBP treated, QC treated and QC/CBP combination treated group. **C.** Graph indicating the excised tumor weight from each of each group (*n* = 10) with cumulative mean; untreated, CBP treated, QC treated and QC/CBP combination treated group. *P < .0028, **P < .0001, treated compared with the untreated group. **D.** Graph showing ascetic fluid volume in control untreated, CBP treated, QC treated and QC/CBP combination treated group. *P < .005 **E.** Graph showing quantitation of Ki67 staining in control untreated, CBP treated, QC treated and QC/CBP combination treated group. *P < .0001 **F.** Measurement of body weight of untreated and treated mice. **G.** TEM analysis of tumor sections obtained from control untreated, CBP treated, QC treated and QC/CBP combination treated group and quantitation *P < .005. (N-Nucleus, AV-autophagosomes and LD-lipid droplets). **H.** Quantitation of # of autophagosomes in untreated control and QC, CBP and QC+CBP treated xenografts (25 images were counted and averaged). **I.** Histology of H and E stained liver from treated and untreated mice.

QC effectively blocked cell growth, induced autophagy and apoptosis in OvCa cell lines *in vitro*. We next tested whether QC in combination with carboplatin is effective in attenuating tumor growth *in vivo*. For this purpose we utilized HeyA8MDR cells that have capacity to form tumors when injected intraperitoneally in nude mice [38]. The effect on tumor growth of QC alone and in combination with carboplatin (CBP in the Figure 6) was evaluated in HeyA8MDR in female nude mouse xenografts. One week after 3×10^6^ HeyA8MDR cells were injected intraperitoneally in the mice, they were randomized into four groups of 10 mice when the tumors were palpable and then treated as shown in Figure 7A. Quinacrine (150 mg/kg body weight) was given as described in the materials and methods section. Quinacrine alone effectively reduced tumor volume and ascites formation (Figures 6B, 6C and 6D). Although carboplatin treatment similarly reduced tumor volume, it was associated with accumulation of ascites to an extent even greater than untreated controls (Figure 6D). Assessment of tumor weight and ascites volume as measured at the time of necropsy across treatment groups showed that combination treatment was more effective in reducing cancer progression compared to all other treatment groups (Figures 7B, 7C and 7D). Staining of the tumors with Ki-67 showed significantly reduced proliferation in QC and QC plus carboplatin treated groups, highlighting the ability of QC to reduce tumor cell proliferation *in vivo* (Figure 7E). There was no significant body weight loss in the QC or combination treatment groups compared to the untreated control group (Figure 7F). TEM micrographs of the xenografts showed more autophagosomes (AV in red, Figure 7G) in the QC group compared to untreated and carboplatin groups. Combination treatment resulted in significantly more autophagosomes compared to QC alone (Figure 7H). H&E staining of the livers from all four groups (Figure 7I) showed that there was no difference in histology, suggesting that neither QC monotherapy nor combination treatment was toxic to the mice. These data demonstrate that QC plus carboplatin combination treatment leads to significantly enhanced antitumor activity in an OvCa mouse model.

## DISCUSSION

The lack of effective treatment modalities for patients with chemoresistant disease continues to pose a therapeutic challenge in ovarian cancer. There is a growing need to identify therapeutic compounds which could promote efficacy to frontline therapeutic agents such as cisplatin and taxol. In this study, we evaluated the anti-cancer properties of the anti-malarial drug QC and tested its effectiveness in combination with carboplatin in OvCa.

QC treatment effectively caused apoptosis and promoted an autophagic response across a variety of ovarian cancer cell lines *in vitro* as well as *in vivo* in mouse-derived HeyA8MDR xenografts. QC treatment caused robust autophagosome formation, LC3B accumulation and p62 downregulation all of which are hallmarks of autophagy. Our findings suggest that QC-mediated induction of autophagy preceded the induction of apoptosis as the effects on the autophagic proteins LC3B and p62 were observed at earlier time points. Autophagy and apoptosis have been previously shown to coincide temporarily in order to cause drug-induced cell death. In agreement with these findings, we showed that by chemically blocking autophagy using bafilomycin A, QC-mediated apoptosis was significantly attenuated. At the molecular level, QC promoted autophagic clearance of p62 in OvCa cell lines. p62 is a well-known substrate of autophagy and high levels of p62 have previously been associated with chemoresistance [23]. Genetic silencing of p62 expression by shRNA in the chemoresistant HeyA8MDR cancer cells promoted apoptosis and enhanced sensitivity to carboplatin (Figure 6E). Therefore, autophagic clearance of p62 expression by QC might be one of the underlying mechanisms responsible for enhanced synergy when QC is combined with carboplatin. Several studies have shown that p62 expression is elevated in breast, pancreatic, colon as well as ovarian cancer which is consistent with our observation of increased expression of p62 in the chemoresistant cell lines that we studied, namely the C13 and HeyA8MDR cell line compared to their chemosensitive counterparts OV2008 and HeyA8. QC treatment down regulated p62 expression in an autophagic-dependent manner in the C13 and HeyA8MDR OvCa cell lines. Lentiviral shRNA-mediated p62 depletion in the OV2008 and HeyA8MDR OvCa cell lines resulted in enhanced synergy between QC and carboplatin. This data suggests that combination treatment with carboplatin and QC results in an enhanced QC-mediated clearance of p62 via autophagy ultimately favoring apoptosis. This observation is further supported by our data showing that inhibition of autophagy by bafilomycin A not only effectively blocked QC-mediated p62 downregulation but also apoptosis. Being a lysosomotropic agent, it is not surprising that QC appears to be implicated in autophagy; however, our study highlights the fact that p62 downregulation plays a key role in QC-mediated apoptosis in OvCa cells and provides a description of the mechanistic interplay between autophagic and apoptotic cell death upon QC treatment.

QC treatment of mouse-derived xenografts was shown to be effective in reducing tumor weight, reducing cell proliferation as measured by Ki-67 staining and blocking ascitic fluid formation. While a similar degree of tumor reduction was achieved by carboplatin treatment alone, in contrast to QC treatment, carboplatin treatment induced ascitic fluid accumulation. TEM analysis of QC-treated xenografts clearly showed increased formation of autophagosomes and autolysosomes. These effects were dramatic when QC was combined with carboplatin leading to complete remission of tumor growth and reduction in the proliferation index. This data is in accordance with the high degree of synergy observed when chemoresistant OvCa cell lines were treated with carboplatin and QC *in vitro*. Taking into consideration the previously proven ability of carboplatin to upregulate p62 expression along with the knowledge that carboplatin-resistant OvCa cells exhibit increased levels of p62, we hypothesize that co-administration of QC with carboplatin may mitigate p62 expression *in vivo* thereby enhancing the degree of apoptosis.

QC has been shown to stabilize p53, inhibit NF-kB, and cause cell cycle arrest. However, QC-mediated p53 stabilization has been associated with its Nf-kB suppressive activities and not due to genotoxic stress [28]. Our findings revealed that QC induced robust autophagic cell death and synergized with carboplatin treatment. It is likely that multiple cellular pathways including p53 and NF-kB in addition to autophagic p62 downregulation are playing a role in QC- and carboplatin-mediated synergy. Other studies have also demonstrated the synergy between QC and other chemotherapeutic drugs such as Lycopene in breast cancer [44], cedarinib in glioma cells [45], vincristine in an MDR sub-clone of K562 cells [31] as well as other therapeutic compounds [46]. More recently, quinacrine has been shown to reverse erlotinib resistance in non-small lung cancer cells by targeting FACT complex and NF-kB activities [47]. It is important to note that several reports have implicated autophagy as a survival mechanism in cancer cells and have demonstrated that by inhibiting autophagy with chloroquine, tumor growth can be arrested [9]. While this is true at a tumor-specific level [48], we believe that prolonged chemical inhibition of autophagy with different autophagic inhibitors might elicit differential responses than just simply inhibition of autophagy. Notably, chloroquine, in addition to inhibiting autophagy, also stabilizes p53 thereby leading to apoptosis. Similarly, another autophagy inhibitor, 3-MA, has been shown to inhibit Akt activation [49]. These observations in conjunction with our data indicate that drugs altering autophagy may also influence apoptosis. QC unlike Bafilomycin A promoted p62 degradation as a function of autophagy induction and also promoted apoptosis both *in vitro* and *in vivo*.

In summary, the present study demonstrated that QC treatment resulted in autophagic clearance of p62, promoted apoptosis and effectively synergized with carboplatin *in vivo*. QC's ability to inhibit multiple pathways simultaneously in addition to promoting autophagic cell death offers compelling evidence to support the hypothesis that it may represent an important adjunct to standard treatment against ovarian cancer especially in patients with chemoresistant disease.

## MATERIALS AND METHODS

### Cell lines, chemicals and antibodies

The human OvCa cell lines SKOV3, SKOV3 TR, HeyA8 and HeyA8 MDR were obtained on an MTA from MD Anderson Cancer Center, Houston, TX. C13 and OV2008, were obtained on a MTA from Dr. Barabara Vanderhyden (Ottawa Hospital Research Institute, Ottawa, Canada). OVCAR3 and CAOV3 were obtained from the American Type Culture Collection (ATCC) (Manassas, VA). All cell lines were cultured according to the providers’ recommendations at5% CO_2_ and at 37°C. Quinacrine was obtained from Sigma-Aldrich. MTT dye was obtained from Promega. Carboplatin was purchased from Calbiochem (San Diego, CA). Anti-LC3B, anti-PARP, anti-p62, anti-PDI and anti GAPDH antibodies were purchased from Cell Signaling Corporation. Anti-NBR antibody was purchased from Genetex. Anti-p53 (DO-1) antibody is from Santa Cruz Biotech.

### Colony formation assay

500 cells were plated in 6-well plates and treated with increasing concentrations of QC (0.12, 0.25, 0.5, 1.0 1nd 2.0, 2.0 μM) for 24 hrs. The media was replaced after day1. Colonies were fixed in methanol and stained with 0.5% crystal violet [50] on day 8 and counted using a colony counting software, Quantity One (Bio-Rad). Each treatment was carried out in triplicate and repeated twice.

### Cytotoxicity assay

MTT assay was performed in order to assess the effect of QC on OvCa cell lines. Ten thousand cells from each cell line were treated with various concentrations of QC and carboplatin separately or in combination for 48 hours followed by a 4-hour period of incubation with 3-(4,5-dimethylthiazol-2-yl)-2,5-diphenyltetrazolium bromide (MTT). The violet formazan crystals were dissolved in dimethyl sulfoxide (DMSO) and the absorbance was measured at 490 nm in a microplate reader.

### ShRNA transductions

The control shRNA, pTRC1-NTC (non-target shRNA vector, Sigma) contains a hairpin insert that will generate siRNAs but contains five base pair mismatches to any known human gene. shRNA for p62/SQSTM denoted as p62-sh280 was purchased from Sigma and with a sequence CGAGGAATTGACAATGGCCAT targeting cDNA region of p62. Lentivirus particles were produced by transient transfection of pTRC1-NTC and pTRC1-p62 shRNA along with packaging vectors (pVSV-G and pGag/pol) in 293T cells. The lentiviral supernatants were collected 48 hours after transduction, filtered and either used for infection or stored at −80°C. Vector titers were determined by transducing OvCa cells with serial dilutions of concentrated lentivirus, in complete growth medium containing 8 μg/ml polybrene (Invitrogen). After 7 days, the growth medium was supplemented with puromycin (2 μg/ml) for selection. The surviving colonies were counted under the microscope and the titer of lentiviral stocks was calculated using the formula: Transducing units = number of colonies × lentiviral dilution. All lentiviral stocks used in the study were selected at a multiplicity of infection of 10.

### MTT assay, synergy assessment and Chou-Talalay calculations

Cell lines were treated with a wide range of concentrations of QC and cisplatin for 48 hours and the half maximal inhibitory concentration (IC_50_) of each drug alone was derived experimentally by MTT assay as previously described [51] and calculated by Prism (GraphPad Software, La Jolla, CA). Subsequently, drug combination studies (QC with cisplatin and QC with carboplatin) were performed and their synergy was quantified using the Chou-Talalay method as previously described in the literature [52, 53]. Both constant ratio synergy (ratio of drugs 1:1) studies were carried out. Synergy was assessed by creating combination indices (CI): CI values less than 0.9 indicate synergism, CI values between 0.9 to 1.1 indicate nearly additive effect and CI values greater than 1.1 indicate antagonism [52].

### Flow cytometric analysis of apoptosis and autophagy

C13 and OV2008 cells were treated with the indicated doses of QC, then harvested and resuspended in a binding buffer. Cells were then stained with Annexin V-pacific blue and Propidium iodide (BD bioscience) according to the manufacturer's instructions. Cells were analyzed in a Beckman Coulter EPICS XL/MCL flow cytometer (Beckman-Coulter Fullerton, CA, USA) and the data were analyzed with Flowjo Software (Tree Star, Ashland, OR, USA). Cyto-ID_TM_ (Enzo Life Sciences) is a dye specifically labeling the autophagic vacuoles in a cell by colocalizing with LC3B. After treating the cells with various concentrations of QC, cells were collected and stained using Cyto-ID dye according to the manufacturer's instructions. Cells were analyzed as previously described in a Beckman Coulter EPICS XL/MCL flow cytometer (Beckman-Coulter Fullerton, CA, USA) and the data were analyzed with Flowjo Software (Tree Star, Ashland, OR, USA).

### Western blot analysis

Western blot analysis was performed as previously described [51, 54].

### Transmission electron microscopy

Cells (2 × 10^6^) were treated with QC and then harvested and centrifuged at 1200 r.p.m. for 5 minutes. Cell samples were then pre-fixed with Trumps buffer. The images were taken using a Philips 208S electron microscope (FEI Corporation, Eindhoven, Netherlands).

### Immunofluorescence

Cells were plated on coverslips and treated with QC as indicated in materials and methods. Cells were fixed with 100% methanol followed by blocking with 1% BSA in PBS. Cells were then incubated with the corresponding primary antibodies at room temperature for 1 hour followed by three washes with 1X PBS and then incubated in the dark with Alexa fluor rabbit anti-mouse (593 nm) in 1% BSA in PBS. Coverslips were washed three times before mounting with Prolong Gold Antifade reagent (Invitrogen). Stained samples were visualized using a Zeiss-LSM 510 fluorescence microscope.

### Animal studies

Athymic nude mice were purchased from Harlan. 3 × 10^6^ HEYA8MDR cells were injected intraperitoneally. Treatment was initiated after one week following the intraperitoneal inoculation of the cells. Stock solutions of QC (Sigma) were prepared in sterile water and administered by oral gavage. Carboplatin (Hospira pharma) were injected intraperitoneally. Before initiation of treatment, animals were randomly assigned to one of four groups (10 mice per group). The control group received water by oral gavage the QC only group received 150 mg/kg body weight of QC every other day starting on day 7, the carboplatin group received carboplatin by intraperitoneal injection at a dose of 50 mg/kg body weight) on days 7, 14, 21 and 28 and the combination group received carboplatin by intraperitoneal injection at days 7, 14, 21 and 28 and QC at 150 mg/kg body weight starting on day 7, then every other day until the end of the study. All animals were sacrificed on day 28. Animal care and procedures was conducted according to institutional policies and the experimental protocol was approved by the IACUC committee of our Institution.

### Statistical analysis

All results are expressed as mean ± standard deviation (S.D.). Data were obtained from three independent experiments. All statistical analyses were conducted using Graph pad Prism software (San Diego, CA). Data were analyzed using paired t test, and P values less than 0.05, unless mentioned otherwise, were considered statistically significant.

## SUPPLEMENTARY MATERIALS FIGURES


