# Supplementary material for: Quinacrine promotes autophagic cell death and chemosensitivity in ovarian cancer and attenuates tumor growth
Source: Oncotarget. 2015 Oct 16;6(34):36354–69. doi: 10.18632/oncotarget.5632 (PMC4742182; doi:10.18632/oncotarget.5632)
Supplement: Supplementary file 1 [file oncotarget-06-36354-s001.pdf]

SUPPLEMENTARY FIGURES

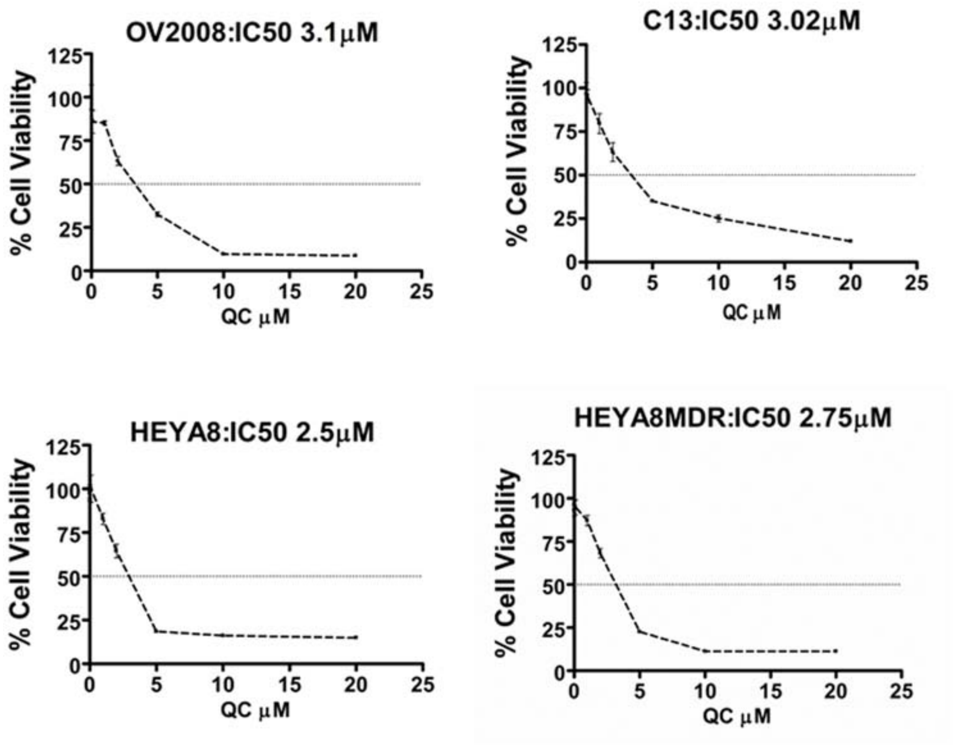

Supplementary Figure S1: Cell viability was determined in OV2008, C13, HeyA8 and HeyA8MDR cells treated with or without QC at indicated concentrations for 24 hours followed by MTT assay.

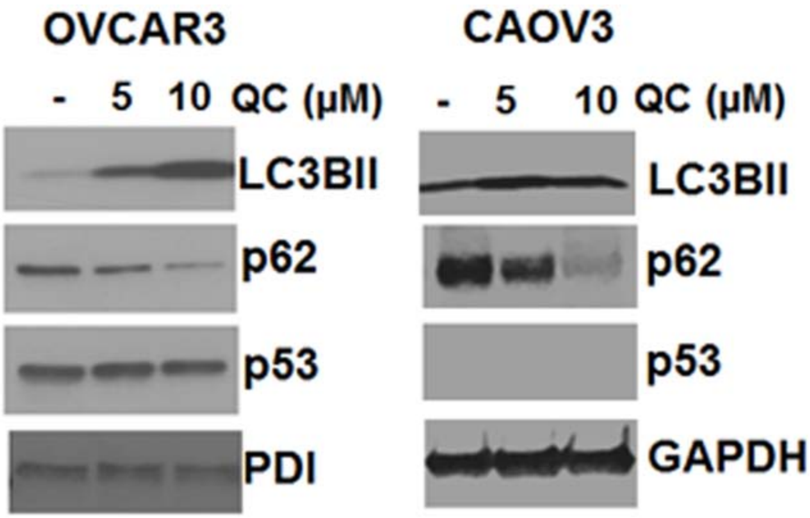

Supplementary Figure S2: Immunoblotting analysis of QC treated high grade serous cell lines, OVCA3 and CAOV3 with anti-LC3B, anti-p62, anti-p53, anti-PDI and anti-GAPDH antibodies.

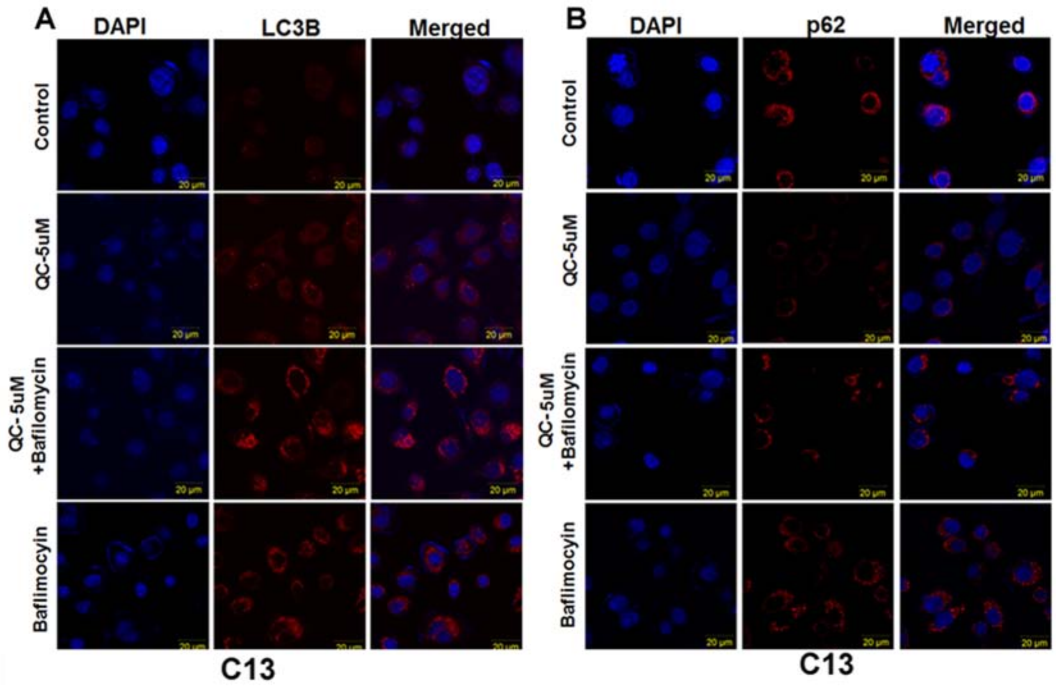

Supplementary Figure S3: Autophagic flux experiment was conducted in C13 cells. Cell were either treated with QC and/or co-treated with bafilomycin A for 12 hours. Cells were fixed and subjected to Immunofluorescence analysis using **A.** anti-LC3B, and **B.** anti-p62 antibodies followed by staining with anti- rabbit TRITC antibody and visualized under confocal microscope. DAPI was used to stain nuclei. QC downregulates LC3B and p62 levels as shown in panels 2 in A and B which is rescued by bafilomycin treatment (Panels 3 and 4 in A and B).

A

Figure S4	A Cisplatin		B Carboplatin		C Cisplatin		D Carboplatin	
	OV2008	C13*	OV2008	C13*	SKOV3	SKOV3TR	SKOV3	SKOV3TR
FA	0.25	0.25	0.25	0.25	0.25	0.25	0.25	0.25
CI	1.268	0.625	1.253	0.693	2.059	0.58	1.466	0.265
s.d.	1.0974	0.281	0.3449	0.1735	0.817	0.2855	0.4045	0.0864
FA	0.5	0.5	0.5	0.5	0.5	0.5	0.5	0.5
CI	1.234	0.642	1.264	0.683	0.912	0.3	1.039	0.356
s.d.	0.5655	0.206	0.2717	0.1197	0.197	0.1015	0.2356	0.0876
FA	0.75	0.75	0.75	0.75	0.75	0.75	0.75	0.75
CI	1.303	0.664	1.281	0.676	0.764	0.186	0.758	0.482
s.d.	0.3819	0.182	0.2446	0.1117	0.1498	0.0621	0.1823	0.1101
FA	0.9	0.9	0.9	0.9	0.9	0.9	0.9	0.9
CI	1.523	0.693	1.305	0.671	0.789	0.131	0.568	0.662
s.d.	0.8266	0.231	0.2792	0.1526	0.1746	0.0673	0.178	0.397

Compare CI values at 75%FA in sensitive (Blue circle) vs resistant (Red circle) cells

B

	IC50 (µM)		
Cell Lines	QC	Cisplatin	Carboplatin
OV2008	3.1	4.4	108
C13	3.02	40	453
HeyA8	2.5	4.8	150
HeyA8MDR	2.75	12.5	200
SKOV3	8	8	355
SKOV3TR	8	16	200

Supplementary Figure S4: To determine whether QC will synergize with cisplatin and or carboplatin, constant ratio synergy studies were performed using the Chou-Talaly method. A. The Combination Index (**CI**) for fractions affected (**FA**) are shown in the table at right **(s.d.**-standard deviation) was determined using the CalcuSyn software. Of note, synergy was demonstrated in the resistant cells across nearly the entire range of the drug concentrations. In contrast, the combination of QC with carboplatin in OV2008 cell line was less synergistic and more additive (S4A and B, columns 1 & 3). Similar studies with isogenic Taxol- sensitive SKOV3 and Taxol-resistant SKOV3TR cells indicate that QC has more synergistic anti-proliferative effects *in vitro* with cisplatin and carboplatin in SKOV3TR (S4C and D, columns 6 & 8) compared to SKOV3 cells (S4C and D, columns 5 & 7). Consistent with this trend, chemo-resistant HeyA8MDR cells showed more synergy with the carboplatin and QC combination compared to the parental chemo-sensitiveHeyA8 cells (Data not shown). As an example, the CI values at 75% FA are indicated by a red circle for the resistant cells compared to isogenic sensitive cells in blue circles. **B.** IC50 values in µM for QC, cisplatin and carboplatin in the paired cell lines used in this study.
